# EFFECTS OF MENOPAUSE AND MARITAL TRANSITIONS ON SUBJECTIVE SEXUAL SELF-EFFICACY AMONG MIDDLE-AGED AND OLDER ADULTS

**DOI:** 10.1093/geroni/igad104.1713

**Published:** 2023-12-21

**Authors:** Melissa Ferguson, Yin Liu

**Affiliations:** Utah State University, Logan, Utah, United States; Utah State University, Logan, Utah, United States

## Abstract

Circumstances in aging including menopause, physical decline, widowhood, divorce and remarriage may influence sexual experiences and well-being in later life. While marital transition can affect both men and women in middle and older life, menopausal transition is especially salient for aging women. This study aims to examine how subjective sexual self-efficacy changes over time and whether marital transitions and menopausal transitions moderate the trajectories. Participants completed two waves of the Midlife in the United States (MIDUS) surveys (N = 1002), ranging from 40 to 84 years of age at baseline (M = 57.44; SD = 10.17) with over half being female (54%, n = 538). Factor analysis confirmed a one-factor model with a five-item subjective sexual self-efficacy construct. Over 10 years, 12% of participants experienced marital transitions, and 29% of women reporting menstrual status experienced menopause transition. Growth curve analysis was used the examine the research questions.

